# The Chikungunya Epidemic on La Réunion Island in 2005–2006: A Cost-of-Illness Study

**DOI:** 10.1371/journal.pntd.0001197

**Published:** 2011-06-14

**Authors:** Man-Koumba Soumahoro, Pierre-Yves Boelle, Bernard-Alex Gaüzere, Kokuvi Atsou, Camille Pelat, Bruno Lambert, Guy La Ruche, Marc Gastellu-Etchegorry, Philippe Renault, Marianne Sarazin, Yazdan Yazdanpanah, Antoine Flahault, Denis Malvy, Thomas Hanslik

**Affiliations:** 1 Université Pierre et Marie Curie-Paris 6, UMR_S 707, Paris, France; 2 INSERM, U 707, Paris, France; 3 Département d'Epidémiologie-Recherche Clinique, Institut Pasteur de Côte d'Ivoire, Abidjan, Côte d'Ivoire; 4 Assistance Publique Hôpitaux de Paris, Service de Santé Publique, Hôpital Saint-Antoine, Paris, France; 5 CHR de La Réunion, Site Centre Hospitalier Félix Guyon, Saint-Denis, La Réunion, France; 6 IMS-Health, Puteaux, France; 7 Institut de Veille Sanitaire, Saint-Maurice, France; 8 CIRE Océan Indien, La Réunion, France; 9 Equipe Avenir ATIP U995, Lille, France; 10 École des Hautes Etudes en Santé Publique, Rennes, France; 11 Université Bordeaux 2, Service de Maladies Infectieuses et Tropicales, Hôpital Saint-André, Bordeaux, France; 12 Assistance Publique Hôpitaux de Paris, Service de Médecine Interne, Hôpital Ambroise Paré, Boulogne Billancourt, France; 13 Université Versailles Saint Quentin en Yvelines, Versailles, France; Centers for Disease Control and Prevention, United States of America

## Abstract

**Background:**

This study was conducted to assess the impact of chikungunya on health costs during the epidemic that occurred on La Réunion in 2005–2006.

**Methodology/Principal Findings:**

From data collected from health agencies, the additional costs incurred by chikungunya in terms of consultations, drug consumption and absence from work were determined by a comparison with the expected costs outside the epidemic period. The cost of hospitalization was estimated from data provided by the national hospitalization database for short-term care by considering all hospital stays in which the ICD-10 code A92.0 appeared. A cost-of-illness study was conducted from the perspective of the third-party payer. Direct medical costs per outpatient and inpatient case were evaluated. The costs were estimated in Euros at 2006 values. Additional reimbursements for consultations with general practitioners and drugs were estimated as €12.4 million (range: €7.7 million–€17.1 million) and €5 million (€1.9 million–€8.1 million), respectively, while the cost of hospitalization for chikungunya was estimated to be €8.5 million (€5.8 million–€8.7 million). Productivity costs were estimated as €17.4 million (€6 million–€28.9 million). The medical cost of the chikungunya epidemic was estimated as €43.9 million, 60% due to direct medical costs and 40% to indirect costs (€26.5 million and €17.4 million, respectively). The direct medical cost was assessed as €90 for each outpatient and €2,000 for each inpatient.

**Conclusions/Significance:**

The medical management of chikungunya during the epidemic on La Réunion Island was associated with an important economic burden. The estimated cost of the reported disease can be used to evaluate the cost/efficacy and cost/benefit ratios for prevention and control programmes of emerging arboviruses.

## Author Summary

For a long time, studies of chikungunya virus infection have been neglected, but since its resurgence in the south-western Indian Ocean and on La Réunion Island, this disease has been paid greater amounts of attention. The economic and social impacts of chikungunya epidemics are poorly documented, including in developed countries. This study estimated the cost-of-illness associated with the 2005–2006 chikungunya epidemics on La Réunion Island, a French overseas department with an economy and health care system of a developed country. “Cost-of-illness” studies measure the amount that would have been saved in the absence of a disease. We found that the epidemic incurred substantial medical expenses estimated at €43.9 million, of which 60% were attributable to direct medical costs related, in particular, to expenditure on medical consultations (47%), hospitalization (32%) and drugs (19%). The costs related to care in ambulatory and hospitalized cases were €90 and €2000 per case, respectively. This study provides the basic inputs for conducting cost-effectiveness and cost-benefit evaluations of chikungunya prevention strategies.

## Introduction

Chikungunya virus infection is an arbovirus infection caused by an Alphavirus of the family *Togaviridae*. This RNA virus is transmitted to humans by mosquitoes of the genus *Aedes*, primarily *Aedes albopictus* and *Aedes aegypti*.

Since 2005, the south-western Indian Ocean has seen the emergence of large-scale epidemics of chikungunya, causing millions of cases in some countries [1]–[5]. In fact, 2005 and 2006 were characterized by a particularly intense spread of the virus. The chikungunya epidemic on La Réunion involved about a third of the population. During this outbreak, the surveillance system estimated that 266,000 cases occurred [6]–[7]. This estimate was validated by a seroprevalence survey conducted after the epidemic [8].

Chikungunya also affected other islands in the Indian Ocean: Mayotte (involving about 38% of the population) [5]–[6], the Grande Comoros (involving about 27% of the population) [9], Madagascar, the Maldives [10], Mauritius [1], [11] and the Seychelles [11]. In India, more than 1.4 million cases were reported in 2006 [12]. Pakistan, Sri Lanka, Malaysia and Indonesia, where chikungunya is endemic, were also affected [11]. Other regions of the world are vulnerable to the spread of this virus or its vector [13], including continental Europe. The risk of local transmission in these countries is not simply theoretical, as shown by the epidemic of chikungunya in the region of Emilia-Romagna, Italy, in 2007 [14], and the detection of two autochthonous cases in south-eastern France in 2010 [15].

The clinical presentation of the disease is characterized by sudden onset fever, accompanied by disabling arthralgia and a skin rash. These signs and symptoms may be accompanied by myalgia, headache, digestive disorders and minimal haemorrhagic and cutaneous manifestations in the form of dyschromia [4], [16]. The signs of the disease generally fade after a few days, but in some cases may persist for several months, particularly regarding rheumatological manifestations [17]–[22]. Severe forms were also described during the epidemic that raged on the island of La Réunion in 2005–2006, which in some cases were associated with death [16], [23]–[35]. A study conducted in the general population at the end of the epidemic on La Réunion showed a seroprevalence of 38% [8]. Almost 85% of infections were symptomatic [36].

The medical economic burden of chikungunya virus infection was recently studied in India, and showed the major impact of this disease on household finances in the absence of medical insurance [37]–[39]. To the best of our knowledge, the economic impact of an epidemic of chikungunya has never been measured in a country with a high level of resources.

The French health care system is based on a universal “social security” system funded by the government, employers and the working population. For historical reasons, people are insured against the risk of disease by schemes that are classified according to their profession: general scheme (most employees, students, recipients of certain benefits and ordinary residents), special scheme (certain categories of civil servants), agricultural scheme (farmers and agricultural workers) and autonomous scheme (artisans, merchants, industrials and liberal professions).

The social security health insurance covers the cost of general and specialized medicine consultations, drugs prescription, laboratory analyses and hospitalization. In the case of sickness, it also provides daily allowances to those who are insured and who are unable to work. Private health insurances may be subscribed to reimburse health related costs not covered by the social security. For the most disadvantaged, State run programs provides universal health coverage.

The objective of this study was to assess the medical costs of the chikungunya epidemic on La Réunion, a French overseas department located in the Indian Ocean, during the period 2005–2006, from a third payer perspective.

## Methods

### Estimation of outpatient medical costs

The direct medical costs of outpatients were defined as general practice consultations, drugs prescription and chikungunya virus specific serological tests. Data were provided by the social security regional health insurance fund of La Réunion and concerned the general and agricultural schemes (75% of the island population).

The choice of drug classes used in this analysis was based on data in the literature [3]–[4], [40]–[42]. The treatments most frequently reported for disease-related symptoms [3]–[4], [40], [42]–[44] essentially involved analgesics and antipyretics. Since the use of non-steroidal anti-inflammatory drugs has been regularly proposed for controlling the often severely painful manifestations of chikungunya infection, reimbursement of the use of proton pump inhibitors was also included in the analysis. Chloroquine and synthetic antimalarials were incorporated because of their indications in the management of certain forms of inflammatory rheumatism, but also because of the initial presumption of their efficacy in the management of the acute phase of chikungunya infection in the context of the epidemic on La Réunion [45]. Lastly, because of the existence of neuropsychiatric manifestations reported in the acute phase of this infection and subsequently [32], [46], anxiolytics were also included in this analysis.

The number of consultations and the drugs costs related to the chikungunya epidemic were estimated from excesses observed during the epidemic period.

Chikungunya serological tests were all attributed to the epidemic as these were not used before the outbreak of chikungunya on the island.

**Figure 1:**
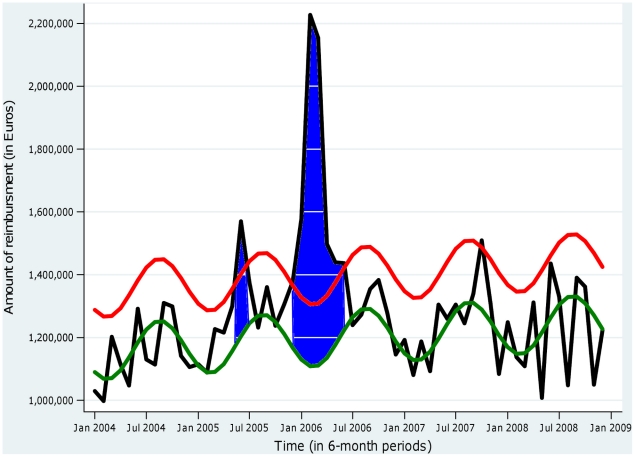
Excess reimbursement of analgesics during the Chikungunya epidemic on La Réunion, 2005–2006. The black curve represents the observed reimbursement costs in Euros, and the green curve the “expected” reimbursement cost in the absence of epidemic, derived from the fit of a periodic regression model to observed costs outside the epidemic period. The red curve represents the upper limit of the 95% prediction interval for monthly costs in the absence of epidemic. Excess periods are defined when the observed costs are above the threshold (area in blue) and quantified by the cumulated difference between observed and expected costs over such periods.

In order to estimate the excess consultation and drug costs due to the epidemic, we first determined what would have been observed in the absence of an epidemic by using a periodic regression model [47]–[48]. For this approach, the observed number of consultations (or level of drugs costs) Y_t_ at time t in the absence of an epidemic is assumed to randomly fluctuate around an expected value m(t). The expected value m(t) was expressed as a periodic function account for seasonal effects, and estimated by least squares fitting to Y_t_ over the non-epidemic period (defined as before March 1^st^, 2005 and after June 30^th^, 2006 [6], [49]). This “expected” number in the absence of an epidemic, shown as a green curve in Figure 1 for analgesics consumption, was estimated for each quantity (consultation, antimalarials, proton pump inhibitors, anxiolytics). An upper threshold, shown as the red curve in Figure 1, was computed as the upper limit of the 95% prediction interval (m(t)+1.65 σ, where *σ* was the residual standard deviation of the regression). Excess periods, shown as blue areas in Figure 1, were define as periods when the observations (number of consultations or drug costs) were above this upper threshold (i.e. Y(t)>m(t)+1.65 σ). The cumulated excess in consultations (or costs) were quantified by cumulating differences between observed and expected (Y_t_ – m(t)) during such excess periods. A lower bound for the excess was calculated by cumulating differences only above the threshold (i.e. Yt – m(t) – 1.65 σ) instead of above the expected value, and an upper bound was obtained by cumulating differences over the whole epidemic period instead of over the excess period. These values are reported as a range to illustrate uncertainty on the estimates. To assess the reproducibility of the approach, an independent estimate of analgesics consumption was obtained by analysing the number of boxes sold by pharmacists (data IMS-Health) during the period 2002 to 2008 (rather than reimbursements from the social security).

The cost of consultations due to chikungunya was estimated as excess number of consultation times the mean social security rate of one consultation (€26.4 in La Réunion).

### Estimation of hospitalization costs associated with the chikungunya epidemic

The cost of the hospitalizations associated with chikungunya was derived from the national database of hospital stays in short-term care (PMSI database) [50]. All hospital stays between March 1^st^ 2005 and June 30^th^ 2006 with ICD-10 code A92.0 (“chikungunya fever”) were included.

In France, the cost of hospitalization is determined on a Diagnosis-Related Groups (DRG) basis [51]–[52]. The classification of a patient in a given DRG is determined according to the final diagnosis and management.

**Figure 2:**
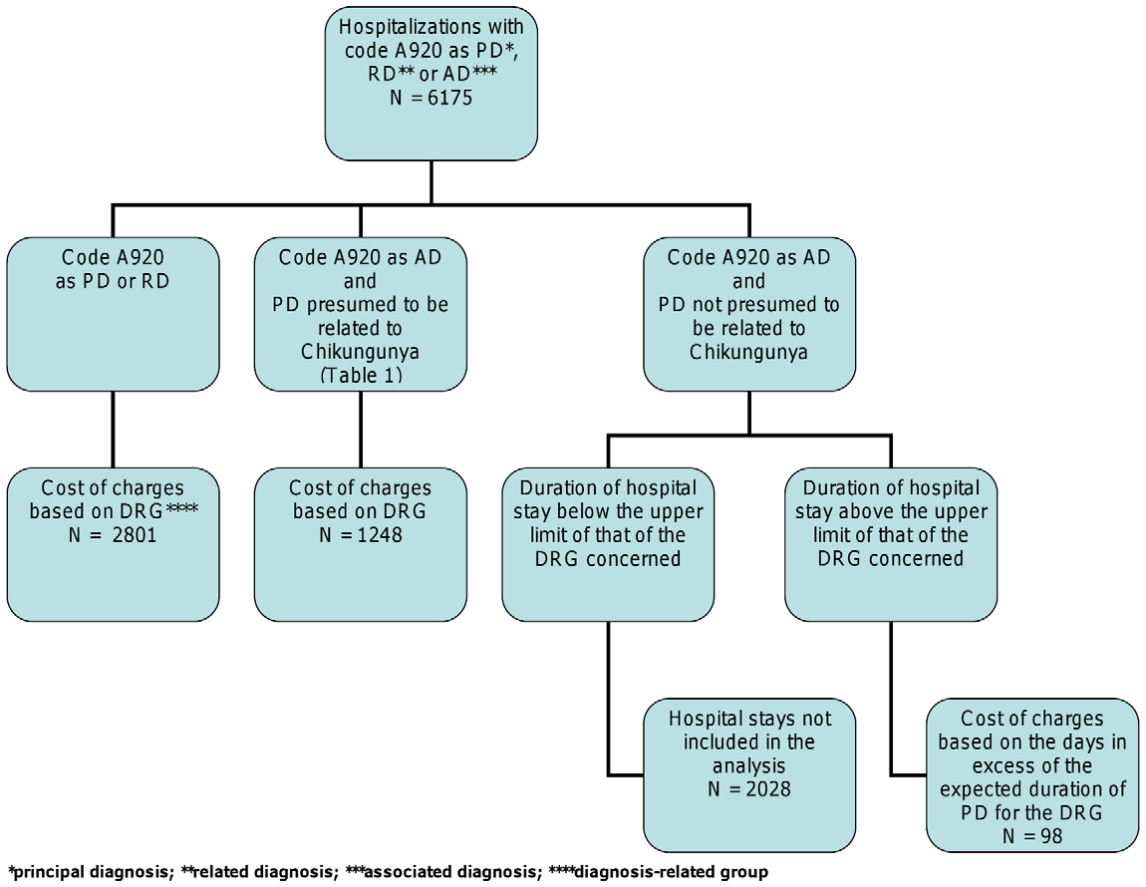
Algorithm for the scale of charges for hospital stays associated with Chikungunya.

Here, the cost of a hospital stay was entirely attributed to chikungunya when the code A92.0 appeared i) either as a principal diagnosis (PD) or as a related diagnosis (RD) or ii) as an associated diagnosis (AD) with a PD consistent with symptoms reported in the acute phase of the disease (the codes concerned are reviewed in Table 1) [4], [16], [53]. For hospital stays where chikungunya was coded as an AD with a PD not consistent with a manifestation of the acute stage of chikungunya, we only took into account the cost of days in excess to the length of stay for this DRG, under the assumption that chikungunya would lengthen the hospital stay (Figure 2). In order to determine the lower estimate of the hospitalization costs related to chikungunya, only stays with A92.0 coded as PD or RD were considered; an upper limit was calculated by including all hospital stays with a chikungunya code (PD, RD and AD, irrespective of the length of stay for the latter). A previous study showed the absence of long-term consequences on medical consumption, so that only acute manifestations were considered [22].

**Table 1:** ICD-10 codes of signs that may be related to Chikungunya virus infection.

ICD-10* chapters and groups of conditions concerned	ICD-10 code concerned
**Chapter I: Certain infectious and parasitic diseases**	
Intestinal infectious diseases	A08, A09
Other bacterial diseases	A40, A41, A46
Viral infections of the central nervous system	A83, A86
Arthropod-borne viral fevers and viral haemorrhagic fevers	A94
Viral infections characterized by skin and mucous membrane lesions	B09
Viral hepatitis	B17, B19
Other viral diseases	B34
**Chapter III: Diseases of the blood and blood-forming organs and certain disorders involving the immune mechanism**	
Coagulation defects, purpura and other haemorrhagic conditions	D69
Other diseases of blood and blood-forming organs	D72, D762
**Chapter IV: Endocrine, nutritional and metabolic diseases**	
Metabolic disorders	E86
**Chapter V: Mental and behavioural disorders**	
Mood disorders	F32
**Chapter VI: Diseases of the nervous system**	
Inflammatory diseases of the central nervous system	G04, G05
Episodic and paroxysmal disorders	G40.9, G43.9
Diseases of myoneural junction and muscle	G72.4
Other disorders of the nervous system	G93.3
**Chapter XII: Diseases of the skin and subcutaneous tissue**	
Infections of the skin and subcutaneous tissue	L08
Bullous disorders	L13, L14
Dermatitis and eczema	L29, L30
Urticaria and erythema	L54.8
**Chapter XIII: Diseases of the musculoskeletal system and connective tissue**	
Arthropathies	M01.8, M06, M13, M25
Soft tissue disorders	M65.8, M63.8, M79
**Chapter XV: Pregnancy, childbirth and the puerperium**	
Other obstetric conditions, not elsewhere classified	O98.5, O99.8
**Chapter XVI: Certain conditions originating in the perinatal period**	
Foetus or newborn affected by maternal factors and by complications of pregnancy, labour and delivery	P00.2
Disorders related to length of gestation and foetal growth	P05**, P07**
**Chapter XVII: Congenital malformations, deformations and chromosomal abnormalities**	
Other congenital malformations	Q81.9
**Chapter XVIII: Symptoms, signs and abnormal clinical and laboratory findings, not elsewhere classified**	
Symptoms and signs involving the digestive system and abdomen	R11
Symptoms and signs involving the skin and subcutaneous tissue	R21
Symptoms and signs involving the nervous and musculoskeletal systems	R29.8
General symptoms and signs	R50, R51, R52, R53, R55**, R56.0
**Chapter XXI: Factors influencing health status and contact with health services**	
Persons encountering health services in circumstances related to reproduction	Z35.8**, Z38.0**

*The International Statistical Classification of Diseases and Related Health Problems 10th Revision.

**Where the code A92.0 was the only AD.

This list of manifestations was compiled following a review of all of the ICD-10 codes by two of the authors (MKS and TH), based on the symptoms reported in the acute phase of the disease. [4], [16], [53].

### Absenteeism costs

Data relating to absenteeism were also collected from the social security regional fund of La Réunion, including the number of days of sick leave from work. The estimate of absenteeism costs due to the chikungunya epidemic was determined from the excess absence observed during the epidemic period according to the method used to evaluate outpatient medical costs, as described above. Thus, a periodic regression model was adjusted for the number of days of absence from work outside the epidemic period (between 2005 and 2008). We used the same method to evaluate the excess number of people who had taken sick leave.

In order to evaluate absenteeism costs, the excess number of days of absence from work was multiplied with the average wage in France. According to data from the INSEE (National Institute for Statistics and Economic Studies), the median wage (which corresponds to the sum of net wages earned by an individual) amounted to €17,000 in 2006. By considering a ratio of 1∶2 between the net salary and gross salary, the annual gross salary amounted to €34,000, which gave a gross daily wage of €155 when considering 220 working days per year on average.

### Cost analysis

In order to evaluate the medical costs related to the epidemic, we performed a cost-of-illness study from the third-party payer perspective. Intangible costs (non-financial costs such as the impact of the disease on quality of life) and non-medical direct costs (transport, home help) were not included in this analysis. Similarly, costs borne by the patient or their private insurance companies were not included.

This estimation established the total direct medical costs (consultations, serological tests, drug consumption and hospitalization) and indirect medical costs (such as disease-related loss of productivity) resulting from all cases of chikungunya during the epidemic on La Réunion.

The direct medical costs were reported for each outpatient case and for each inpatient case. For the outpatients, the database provided by the social security regional fund of La Réunion was that of the general and agricultural schemes which covers 75% of the population of the island (source: social security fund of La Réunion). Therefore, we undertook the analysis by assuming that the data were only related to 75% of the cases of chikungunya (i.e. 199,500 people). For the inpatients, the database included all of the hospitalized cases of chikungunya that had been used to calculate the cost per inpatient.

All of the costs were rounded off to the nearest hundred thousand Euros for the total population and the nearest unit for the cost per case.

All data were analysed using periodic regression software [54] and Stata10.0™ software (StataCorp 2008, Texas, USA). The costs were estimated in Euros at 2006 values.

## Results

The additional number of consultations during the epidemic compared to non-epidemic periods was 470,000 (range = 195,000–765,000), an increase of 25% (range = 16–35%), corresponding to an average of 2 additional consultations per case. The cost of these additional consultations amounted to €12.4 million (Tables 2 and 3).

**Table 2:** Consultations, drug reimbursements and absenteeism from work due to the Chikungunya epidemic, La Réunion, 2005–2006.

Parameter	Proportion of excess* [range]	Quantification of excesses (in thousands) [range]
**Consultations (services)**	25% [16–35%]	470 [195–765]
**Drugs reimbursements (cost in Euros)**		
Antimalarials	59% [54–64%]	36.2 [12.7–61.1]
Analgesics	44% [16–71%]	4027 [2467.1–4720.2]
Proton pump inhibitors	30% [9–50%]	876.5 [432.9–1187.2]
Anxiolytics	24% [0–52%]	43.4 [15–78.5]
**Sick leave**		
Number of people concerned	137% [0–275%]	12.8 [10.7–13.6]
Number of days reimbursed	53% [15–92%]	112.4 [62.4–112.4]

*Compared to consumption outside the epidemic, calculated by a periodic regression model.

**Table 3:** Medical costs related to the Chikungunya epidemic, La Réunion, 2005–2006.

Costs	Parameters	Total cost (in millions of Euros)	Proportion of total cost
**Direct costs**		**26.5**	**60%**
	Consultations	12.4	47%
	Drugs	5	19%
	Serological tests	0.57	2%
	Hospitalizations	8.5	32%
**Indirect costs**		**17.4**	**40%**
	Sick leave	17.4	100%
**Total medical cost**		**43.9**	**100%**

The excess cost for drugs was 59% (54–64%) for antimalarials, 44% (16–71%) for analgesics, 30% (9–50%) for proton pump inhibitors and 24% (0–52%) for anxiolytics, yielding a total excess cost of €5.0 million (Tables 2 and 3).

**Figure 3:**
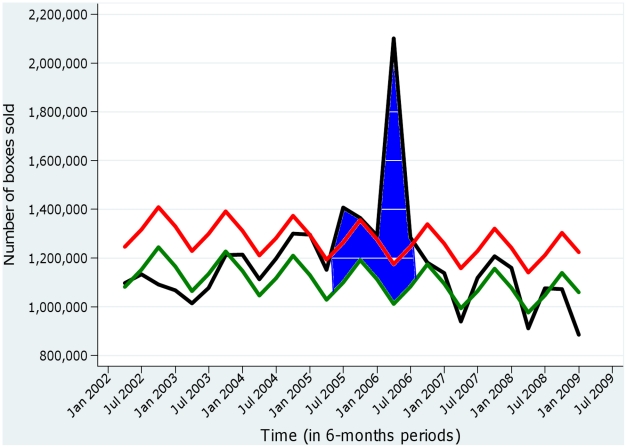
Excess sales of analgesics observed during the Chikungunya epidemic on La Réunion, 2005–2006. The black curve represents the observed number of boxes sold, and the green curve the expected number of boxes sold. The red curve represents the upper limit of the 95% prediction interval. Excesses are represented by the areas painted in blue (source of the data: IMS Health).

Analysis of drug pharmacy sales data also showed an increase of 35% (0–82%) for pain relievers (Figure 3).

The amount of expenditure occasioned by the serological tests was €570,000 for a total number of 29,664 procedures.

The code A920 appeared as the principal, related or associated diagnosis in 6175 hospital stays between March 2005 and June 2006. The inpatient population consisted of 40% men and the mean age was 49±29 years (range: 0–101 years). The code A92.0 appeared as a PD, RD or AD for 2771 (45%), 30 (0.5%) and 3374 (50.5%) patients, respectively. Among the patients for whom this code appeared as an AD, 1248 (i.e. 37%) had a PD for which the ICD-10 code was that of a symptom related to the infection. Based on the algorithm defined in Figure 2, the number of stays included in the estimate of expenses associated with hospitalizations for chikungunya was 4147. The mean duration of hospitalization was 5±7 days (range: 0–146 days) with a median of 3 days, giving a total of 22,134 days. The cost distribution of hospitalization was skewed towards larger values, with a range of €215 to €8000 and, a median at €1600 and, a mean at €2000 per hospitalization. The total cost for all hospitalizations for chikungunya was €8.5 million (€5.8 million–€8.7 million) (Table 3).

Compared with non-epidemic periods, the chikungunya epidemic led to an additional 112,400 (range = 62,400–112,400) days of absence from work for 12,800 (range = 10,700–13,600) subjects, the cost of which was estimated at €17.4 million (Table 3).

The direct and indirect medical costs totalled €43.9 million (Table 3).

Applying this expenditure to subjects affiliated with the general and agricultural social security schemes in La Réunion (i.e. 75% of cases, n = 199,500) only, the cost of outpatient care was estimated as €90 per case for direct costs and €177 per case for all direct and indirect costs.

The mean cost per inpatient case was €2000±€1800 and the mean cost per subject with sick leave was €1360.

## Discussion

This study estimated the medical costs associated with the chikungunya epidemic that occurred in 2005–2006 on La Réunion Island, a French overseas department with the economy and health care system of a developed country. The epidemic incurred substantial medical expenses for the third-party health care payer, estimated as €43.9 million, of which 60% was attributable to direct medical costs related, in particular, to expenditure on medical consultations (47%), hospitalization (32%) and drug consumption (19%).

“Cost-of-illness” types of analysis are interested in the amount that would have been saved in the absence of a disease and which could have been allocated to other sectors. For example, the chikungunya epidemic on La Réunion occasioned greater expenses for the National Health Insurance than occurred for the reimbursement of anti-flu vaccines for the whole of France, estimated in 2006 as being more than €19 million [55].

“Cost-of-illness” studies can help in public health decisions and in the prioritization of health care expenditure by third-party payers. Although they do not take into account the benefits that may be derived from the expenditure they estimate, they are a useful and essential preliminary analysis before cost-efficacy or cost-benefit analyses are undertaken.

To the best of our knowledge, the only published data on an economic evaluation of an epidemic of chikungunya came from surveys conducted in India [38]–[39]. However, the differences in terms of the economic profile and health system organization between La Réunion and India limit the value of a direct comparison of the cost per case. Moreover, it should be noted that epidemiological situations in Asian countries are characterized by recurrent outbreaks with an endemic background, which are very different from those in the Indian Ocean islands where the first emergence of chikungunya was in entirely immunologically naive populations. On the other hand, economic evaluations in economically developed countries have been conducted following epidemics of arboviruses other than chikungunya. This is the case with the Ross River Virus (RRV) epidemic that occurred in Australia in the 1990s. This arbovirus, also caused by an Alphavirus, has a very similar clinical presentation to that observed in chikungunya virus infection. From the data supplied by Harley *et al.* and Mylonas *et al.* [56]–[57], it is possible to estimate that the direct medical costs for outpatient care resulting from the RVV infection were between €61 and €121 per case (figures updated for the year 2006), which are of the same order as those reported in our study for chikungunya. Cost-of-illness studies have also been conducted on other arboviruses such as dengue. For example, a cost-of-illness study was conducted in Cambodia, a country with poor health and economic indicators, in order to determine the cost of dengue. During the 2007 dengue epidemic, the direct medical cost per case was US$29, in which out-of-pocket represented 60% [58]. By reporting the outpatient costs (€90) and inpatient costs (€2000) due to chikungunya as a percentage of the GDP (Gross Domestic Product) per capita of La Réunion (€16,260/inhabitant in 2006), our estimations were found to be considerably higher (0.6% and 12.3% of the GDP, respectively) than those reported by Beauty *et al.* (0.03% and 0.17% of the GDP respectively) in Cambodia [58]. The cost of dengue cases was also estimated in eight countries in the Americas and Asia in a prospective study [59]. The direct medical costs were I$116 for outpatients and I$915 for inpatients (expressed in international Dollars (I$) at 2005 value). However, a comparison with chikungunya is difficult because, on the one hand, dengue can be a much more serious disease and, on the other hand, the health systems and economic contexts in these countries are different from those of France, where the largest share of health expenditure is devoted to public insurance.

During the epidemic period, only a proportion of all of the drug prescriptions was attributable to chikungunya. Using the periodic regression model we were able to determine this contribution to the costs of consultations and drugs, as well as to the daily payments to those on sick leave. Regarding the item relating to the serological tests, these were performed so infrequently on La Réunion before the chikungunya epidemic that all the reimbursements made during the epidemic period were taken into account in the costing.

The excess costs of chikungunya were estimated by subtracting the expected costs in the absence of an epidemic from observed costs. The expected costs were extrapolated from available data outside the epidemic period, under the hypothesis that such costs would be stationary, albeit seasonally varying, from one year to the next. Available data to estimate the expected costs included the beginning of 2005 and years 2007–2008 or, in other words, essentially post-epidemic periods. Visual inspection of the monthly time series did not evoke a marked before/after epidemic change in costs, suggesting that the expected costs were reasonably estimated this way. Concerning the variability of the estimates, our approach was primarily pragmatic, as the main source of uncertainty was how to define excesses rather than statistical variability. The ranges reported are therefore not confidence intervals in the statistical sense, yet illustrate the likely range of excess costs. Since the cost of analgesics accounted for 80% of the drug expenditure related to chikungunya, we checked for a possible bias by analysing the data for drug sales in pharmacies from 2002 to 2008 and found an increase of 35% in the sales of boxes of analgesics during the epidemic period, a proportion similar to that for the increased reimbursement of analgesics found in this study (44%). These data, which are presented in Figure 3, confirm the results of our analysis based on the data of the social security regional health insurance fund of La Réunion.

Our study had some limitations. First, the evaluation of health care expenses did not take into account consultations with specialists (rheumatologists or dermatologists, for example). However, on La Réunion Island, the number of specialists is very small and general practitioners retain a predominant role. Second, the cost of manifestations in the late phase of the disease were not included in our analysis, but we have previously shown that these manifestations did not lead to a significant increase in drug consumption [22]. Third, the estimate of indirect costs reported here did not take into account the fact that social security does not cover an absence from work for fewer than 3 days, which represents a possible source of cost underestimation. Fourth, indirect costs in cost-of-illness analyses often evaluate productivity losses, including costs from the perspective of the patients (and often the caregivers), which was not the case in the present study. As our study was not patient based, it was not possible to assess the costs from the patient's point of view.

The costs of this disease were estimated from the third-party payer's perspective. If the perspective were to be widened, this would increase the estimate of the cost of the disease by including, for example, direct medical costs not reimbursed by social security (self-medication, alternative medicines, the proportion of costs borne by the patient or his/her medical insurance company) and non-medical direct costs (transport costs, childcare costs) and intangible costs (loss of well-being, pain, immobilization).

Fifth, self-medication was certainly part of the spending, but we were not able to find specific data about this. In France, the self-medication market is less well developed than in neighbouring countries (such as Poland, England, Italy, Germany). Indeed, these drugs only represented 6% and 6.5% of all drugs sold in 2006 and in 2009, respectively [60].

On La Réunion, alternative medicines are generally based on the use of products (*zerbages* or herbal tea infusions) that have not had their therapeutic efficacy demonstrated (*Noni* juice, tonics, essential oils) and which are not covered by the National Health Insurance scheme, even though the burden falls on households. These are costs that are difficult to measure retrospectively. Sixth, concerning private insurance, we could not obtain precise information on its coverage in La Réunion. However, social security reimburses a large share of the costs; for the most disadvantaged, the costs are reimbursed in full.

The high cost of management explains the high expenditure involved in combating disease. In fact, the amount of economic assistance provided by the French state for the health crisis of chikungunya, as notified by the general secretariat for regional affairs (SGAR) in La Réunion, was higher than the budget set aside for the direct medical costs of the epidemic. Thus, €31.5 million was spent under the Intervention Fund for the Support of Crafts and Trade (FISAC) and the Exceptional Aid Fund (FSE) (source: Prefecture of La Réunion). The increased activity resulting from the epidemic also incurred costs in hospitals. By 31^st^ March 2006, €11.9 million had been allocated by the La Réunion Regional Hospitalization Agency to cover the costs associated with the additional expenditure on personnel, insect control, hospital equipment and research.

Cost estimates of a disease may be used to evaluate the cost/benefit ratio of monitoring, prevention and control programmes of arboviruses such as chikungunya, whether in the context of La Réunion (where re-emergence remains a possibility) or in other regions of the world (that are vulnerable to the spread of this virus or its vector). Cost estimates will also be essential in evaluating the efficacy of candidate vaccines or future vaccination strategies.
